# Are measures of voice hearing distinct from measures of emotional states, recovery and well-being? A factor analysis study

**DOI:** 10.1371/journal.pone.0333069

**Published:** 2025-10-07

**Authors:** Sofia Loizou, Andy P. Field, David Fowler, Mark Hayward

**Affiliations:** 1 School of Psychology, University of Sussex, Falmer, United Kingdom; 2 Research & Development Department, Sussex Partnership NHS Foundation Trust, Hove, United Kingdom; The Hong Kong Polytechnic University, HONG KONG

## Abstract

**Background:**

Conceptually, there may be some overlap between measures of voice hearing experiences and measures assessing broader outcome domains. Despite this possibility, it is unknown whether measures of voice hearing and broader outcomes are assessing similar or separate concepts. This study aimed to examine whether measures of voice hearing are distinct from measures of emotional states, well-being and recovery.

**Methods:**

Study 1 examined whether the Hamilton Program for Schizophrenia Voices Questionnaire is distinct from the Depression Anxiety Stress Scale-21 and the Short Warwick-Edinburgh Mental Well-being Scale using secondary data (*n* = 401). Study 2 examined whether the Psychotic Symptoms Rating Scale for Auditory Hallucinations is distinct from the Hospital Anxiety Depression Scale and the CHoice of Outcome in Cbt for psychoses short form using baseline data from two randomized controlled trials (*n* = 187).

**Results:**

In Study 1, a six-factor model was found to be reasonable and accounted for 54.04% of the total variance (F1: 13%, F2: 11.26%, F3: 8.55%, F4: 4.04%, F5: 7.30%, F6: 9.9%). In Study 2, a five-factor model was identified and accounted for 39.99% of the total variance (F1: 15.52%, F2: 7.47%, F3: 6.53%, F4: 6.70%, F5: 3.78%). Within both studies, the items from the voice hearing measures loaded uniquely onto factors that contained no items from other measures.

**Conclusion:**

Findings show that measures of voice hearing are distinct from broad outcome measures and therefore are measuring separate concepts. This confirms the psychometric properties of the voice hearing measures and provides some clarity around outcomes and their measurement.

## Introduction

A symptom-specific approach to the treatment of psychotic experiences has been developed in response to the limitations identified in trials of CBTp, such as small-to-medium effect sizes, heterogeneity in presentation and reliance on broad severity outcome measures that may not capture changes generated by CBTp [1,2]. By targeting mechanisms linked to individual symptoms, this approach may reduce heterogeneity and enable the use of outcome measures that are more closely aligned with the aims of CBTp. However, recent studies of psychological interventions for distressing voices have used a range of outcomes, suggesting differing understandings among experts about the changes that may be generated by these interventions [3]. This heterogeneity of outcomes has also impeded the comparability of interventions to determine whether symptom-specific approaches are superior in efficacy to general CBTp [1]. This can create confusion for clinicians and patients as they seek to choose from a range of treatment options.

The confusion for clinicians and patients has been exacerbated by the array of measures that have been used to capture outcomes. In some cases, measures of anxiety and depression (e.g., Depression Anxiety Stress Scale-21 [DASS-21] [4], Hospital Anxiety Depression Scale [HADS] [5]) have been used to index the emotional impact of voices. However, it is unclear whether measures of voice hearing (e.g., Hamilton Program for Schizophrenia Voices Questionnaire [HPSVQ] [6], Psychotic Symptoms Rating Scale – Auditory Hallucinations [PSYRATS-AH] [7]) and emotional distress (i.e., anxiety and depression) capture distinct concepts or whether there is a considerable measurement overlap, given they may contain items capturing similar emotional states. This potential measurement overlap, may also arise from how items are understood or interpreted, and whether patients and/or clinicians ascribe certain experiences specifically to voices or to broader emotional distress. Consistent with this, previous research has shown strong associations between anxiety, depression, and aspects of voice hearing including severity, content, loudness, intrusiveness, distress and perceived power [8–13]. This raises the possibility that changes observed in emotional distress following psychological interventions may in part reflect changes in voice-related distress (and vice versa). This highlights the importance of clarifying the distinctiveness of these measures to avoid redundancy, reduce participant burden of having to complete multiple measures, increase outcome measurement precision and improve the comparability of findings across research and clinical contexts.

Measures assessing broader outcome domains such as recovery (CHoice of Outcome in Cbt for psychoses [CHOICE] [14,15]) and well-being (Short Warwick-Edinburgh Mental Well-being Scale [SWEMWBS] [16,17]) have also been linked to measures of depression, anxiety and psychosis-related distress, supporting the suggestion that there may be an overlap in measurement. While voice hearing, anxiety, depression, recovery, and wellbeing are distinct concepts, the extent to which existing measures substantially overlap or whether they capture distinct dimensions has not been explored. Therefore, it is unclear whether their combined use provides supplemental information, or whether they may, in practice, be capturing overlapping aspects of the same experience due to similarities in item content. Clarifying this relationship is important for ensuring precise and transparent outcome measurement in both research and routine clinical practice.

The purpose of the study was to examine whether voice hearing measures are distinct from measures of emotional states, recovery and well-being by undertaking factor analysis. Two questions were addressed: 1) Is HPSVQ distinct from DASS-21 and SWEMWBS? (Study 1) and 2) Is PSYRATS-AH distinct from HADS and CHOICE-SF? (Study 2). If items from both voice-hearing and broader outcome measures load onto the same factor, this would suggest that measures of voice hearing may not be entirely distinct and instead share substantial variance. If items from the voice hearing measures load uniquely onto their own factors, this would suggest that these measures are capturing distinct concepts.

## Study 1: HPSVQ, DASS-21 and SWEMBS

### Methods

#### Design.

Study 1 used cross-sectional data from existing datasets to determine the factor structure of HPSVQ, DASS-21 and SWEMWBS. The dataset from the Approve study [18] was used to identify underlying factors and the dataset from the Voice Impact Scale study (VIS [19]) was used to verify the factor structure. Data were accessed between 2019–2023. Data were anonymised and therefore individual participants could not be identified.

***Participants.*** Participants (*n* = 401) were recruited from inpatient and mental health NHS services as part of the Approve study and had to meet the following criteria: a) 18 years of age and above, b) experiencing voice hearing for at least six months irrespective of diagnosis and c) have sufficient English language skills. In the VIS study, participants (*n* = 398) were also recruited from mental health NHS services. Participants were: a) at least 18 years of age, b) met diagnostic criteria for schizophrenia- or psychosis-spectrum disorders, c) experienced voices for minimum a year, and d) understood written English. The two samples were statistically comparable on all demographic variables except for the percentage of participants from black and mixed ethnic backgrounds, and the percentage of participants with a diagnosis of schizophrenia (Table 1). S1 Table in S1 File shows the descriptive statistics.

**Table 1:** Demographic information of participants in study 1.

	Approve sample (*N* = 401)	VIS sample (*N* = 398)	t-test/z-test
**M age (*SD*)**	40.4 (13.4)	40.2 (12.8)	*t*(793.28) = 0.30, *p* = .766
**M age voice onset (*SD*)**	22.4 (11.3)	22.8 (10.5)	*t*(749.15) = −0.57, *p* = .571
**M voice duration (*SD*)**	17.98 (13.8)	17.03 (13.0)	*t*(746.58) = −0.97, *p* = .330
**Gender (%)**			
* Male*	244 (60.8)	254 (63.8)	*z* = −0.87, *p* = .384
* *Female	151 (37.7)	134 (33.7)	*z* = 1.18, *p* = .238
* Another term*	2 (0.50)	1 (0.3)	*z* = 0.57, *p* = .568
* Prefer not to say*	1 (0.3)	–	–
**Ethnicity (%)**			
* White*	350 (87.3)	330 (82.9)	*z* = 1.73, *p* = .083
* Asian*	16 (4.0)	22 (5.5)	*z* = −1.02, *p* = .307
* Black*	8 (2.0)	20 (5.0)	*z* = −2.33, *p* = .019
* Mixed*	11 (2.8)	22 (5.5)	*z *= −1.98, *p* = .047
* Other*	14 (3.5)	–	–
* Prefer not to say*	1 (0.3)	1 (0.3)	*z* = −0.005, *p* = .992
**Employment (%)**			
* Employed*	53 (13.2)	43 (10.8)	*z* = 1.05, *p* = .293
* Unemployed*	262 (65.3)	277 (69.6)	*z* = −1.28, *p* = .197
* Student*	21 (5.2)	16 (4.0)	*z* = 0.82, *p* = .412
* Retired*	21 (5.2)	18 (4.5)	*z* = −0.46, *p* = .638
* Homemaker*	6 (1.5)	3 (0.8)	*z *= 0.99, *p* = .322
* Other*	34 (8.5)	21 (5.3)	*z *= 1.78, *p* = .734
* Prefer not to say*	3 (0.7)	9 (2.3)	*z *= −1.76, *p* = .078
**Education (%)**			
* Left school <16*	78 (19.4)	81 (20.4)	*z* = −0.31, *p* = .748
* Left school at 16*	104 (25.9)	119 (29.9)	*z* = −1.25, *p* = .211
* Left school at 17/18*	62 (15.5)	46 (11.6)	*z* = 1.61, *p* = .1074
* College*	76 (18.9)	70 (17.6)	*z* = 0.50, *p* = .617
* University*	77 (19.2)	74 (18.6)	*z* = 0.22, *p* = .825
* Prefer not to say*	4 (1.0)	–	–
**Diagnosis (%)**			
* Schizophrenia*	192 (47.9)	341 (85.7)	*z* = −11.33, *p* < .001
* Schizoaffective*	40 (10.0)	54 (13.6)	*z* = −1.58, *p* = .114
* BPD/EUPD*	14 (3.5)	–	–
* PTSD*	2 (0.5)	–	–
* Depression*	7 (1.7)	–	–
* Mixed*	90 (22.4)	–	–
* Other*	17 (4.2)	–	–
* None found*	36 (9.0)	–	–

*Note*. Approve missing data for age (*n* = 2), age voice onset (*n* = 5), voice duration (*n* = 7), gender (*n* = 3), ethnicity (*n* = 1), employment (*n* = 1) and diagnosis (*n* = 3); VIS missing data for age onset (*n* = 42), gender (*n* = 9), ethnicity (*n* = 3), employment (*n* = 11), education (*n* = 8), diagnosis (*n* = 3).

#### Materials.

**Hamilton Program *for* Schizophrenia Voice Questionnaire (HPSVQ [6]).** The HPSVQ is a 9-item self-report measure developed to assess the characteristics and negative impact of voice hearing. Each item is rated on a 5-point rating scale (0–4), with higher scores reflecting greater severity or impairment. The HPSVQ has been found to have acceptable internal consistency and test-retest reliability [6].

**Depression, Anxiety and Stress Scale – 21 items (DASS-21 [4,20].** The DASS-21 is a self-report measure assessing the severity of emotional states of depression (7 items), anxiety (7 items) and stress (7 items). Items are rated on a 4-point rating scale (0 = *did not apply to me*, 1 = *applied to me to some degree, or some of the time*, 2 = *applied to me to a considerable degree, or a good part of time*, 3 = *applied to me very much, or most of the time*). The DASS-21 has good internal consistency and good concurrent validity [21].

**Short Warwick-Edinburgh Mental Well-being Scale (SWEMWBS [16], WEMWBS [22]).** The SWEMWBS is a 7-item self-report scale assessing well-being. Items are rated on a 5-point rating scale (1 = *none of the time*, 2 = *rarely*, 3 = *some of the time*, 4 = *often*, 5 = *all of the time*). The scale has shown excellent internal consistency in clinical samples [23]. Items have been reverse-coded, so that higher scores reflect lower well-being and are consistent with the HPSVQ and DASS-21 scores.

### Procedure

Eligible service users were referred to the Approve study by clinicians. Those interested in taking part met with a researcher to review the participant information sheet and the consent form. Once written consent was received, service users were asked to complete a questionnaire battery. In the VIS study, service users were identified either by clinicians or through an NHS trust research system. Eligible service users were sent a letter inviting them to take part. Written consent was received for all participants. Consented service users were asked to complete a set of questionnaires. The Approve study (recruitment start date:01/02/2018, recruitment end date: 31/08/2019) received ethical approval by the London-South-East REC and HRA (18/LO/0046) and the VIS study (recruitment start date: 01/06/2016, recruitment end date: 01/01/2017) received ethical approval by the North West – Lancaster REC and HRA (16/NW/0446).

### Statistical analyses

All statistical analyses were carried out in R [24]. Full information maximum likelihood (FIML) was considered as an appropriate method for handling missing data on the basis that the proportion of missing data was low (Approve 0.61%, VIS 2.15%). Parallel analysis [25] and a scree plot were used to determine the number of factors to retain. Parallel analysis compares the observed eigen values of the correlation matrix with those from a random data matrix. Exploratory factor analysis (EFA) was performed on the Approve data to identify the underlying factor structure of the HPSVQ, DASS-21 and SWEMWBS. Oblique rotation was used under the assumption that the factors will be correlated [26]. The ‘psych’ package [27] was used for these analyses. Confirmatory factor analysis (CFA) was performed on the VIS data using the ‘lavaan’ package [28] to verify the factor structure derived from the EFA. The following fit indices were used to examine model fit [29,30]: χ2 to degrees of freedom (χ2/df ratio < 2), Comparative Fit Index (CFI) good fit > 0.95, sufficient fit > .90; Tucker Lewis Index (TLI) good fit > 0.95, sufficient fit > 0.90; Root Mean Square Error of Approximation (RMSEA) close fit < 0.06, adequate fit < 0.08; and Standardized Root Mean Residual (SRMR) good fit < 0.05, sufficient fit < 0.08.

### Results

#### Exploratory factor analysis.

The Kaiser-Meyer-Olkin statistic suggested there were sufficient numbers of variables to determine a factor structure, KMO = .95, and Barlett’s test confirmed that correlations were significantly different to zero, χ2(666) = 8481.53, *p* < .001. The correlation matrix (S2 Table in S1 File) suggested correlations above zero and none were too large to suggest multicollinearity or singularity (i.e., *r* < 0.80). Parallel analysis suggested six factors and the scree plot had points of inflexion at 2, 3, 4, 5 and 6 factors. It was concluded that the six-factor model was reasonable (TLI = 0.953, RMSEA = 0.037, RMSR = 0.02, CFI = 0.967) (S3 Table in S1 File).

The six-factor model accounted for 54.04% of the total variance of the original data (Table 2). Factor 1 (depression) accounted for 13% of the variance and consisted of eight items of the DASS-21, of which seven items related to depression (anhedonia – item 3, inertia – item 5, hopelessness – item 10, dysphoria – item 13, lack of interest – item 16, self-deprecation – item 17, devaluation of life – item 21) and one related to anxiety (situational anxiety – item 9). Factor 2 (voice hearing experience) consisted of eight items of the HPSVQ (frequency – item 1, negative content – item 2, loudness – item 3, duration – item 4, interference with daily activities – item 5, distress – item 6, impact on self-appraisal – item 7, clarity – item 8), and accounted for 11.26% of the variance. Factor 3 (well-being) consisted of all items of the SWEMWBS and explained 8.55% of the variance. Factor 4 (voice characteristics) consisted of five items of the HPSVQ (frequency – item 1, loudness – item 3, duration – item 4, clarity – item 8, obey commands – item 9) and accounted for 4.04% of the variance. Factor 5 (stress) consisted of five items of the DASS-21 relating to stress (difficulties relaxing – items 1 and 12, nervous arousal – item 8, agitation – item 11, impatience – item 14) and 1 item of the SWEMWBS (feeling relaxed – item 3) and accounted for 7.30% of the variance. Factor 6 (states of anxiety and stress) explained 9.9% of the variance and consisted of eight items of the DASS-21, of which six items related to anxiety (autonomic arousal – items 2, 4 and 19, skeletal musculature effects – item 7, subjective anxious affect – items 15 and 20) and two items related to stress (nervous energy – item 8, irritable/over-reactive – item 18). The inter-factor correlations showed small-to-medium correlations between voice-specific (2, 4) and broad factors (1, 3, 5, 6) (S4 Table in S1 File).

**Table 2:** Exploratory factor analysis (HPSVQ, DASS-21 and SWEMWBS).

Items	F1	F2	F3	F4	F5	F6
HPSVQ 1 How frequently did you hear a voice or voices?	0.02	**0.41**	0.00	**0.49**	0.06	−0.07
HPSVQ 2 How bad are the things the voices say to you?	0.01	**0.81**	−0.01	−0.04	0.06	−0.04
HPSVQ 3 How loud are the voices?	−0.07	**0.51**	0.10	**0.30**	0.06	0.07
HPSVQ 4 How long do the voices usually last?	−0.05	**0.51**	0.04	**0.41**	0.04	0.01
HPSVQ 5 How much do the voices interfere with your daily activities?	0.09	**0.63**	0.07	0.13	0.11	0.00
HPSVQ 6 How distressing are the voices that you hear?	0.00	**0.85**	0.02	−0.04	−0.01	0.13
HPSVQ 7 How bad do the voices make you feel about yourself?	0.22	**0.75**	0.04	−0.09	0.00	0.02
HPSVQ 8 How clearly do you hear the voices?	−0.06	**0.55**	−0.02	**0.36**	−0.01	0.00
HPSVQ9 How often do you do what the voices say?	0.18	0.14	0.16	**0.43**	−0.18	0.15
SWEMWBS 1 I’ve been feeling optimistic about the future	0.19	0.04	**0.65**	−0.08	−0.08	0.00
SWEMWBS 2 I’ve been feeling useful	0.13	0.05	**0.59**	−0.13	0.08	−0.07
SWEMWBS 3 I’ve been feeling relaxed	−0.15	0.16	**0.50**	−0.10	**0.37**	0.12
SWEMWBS 4 I’ve been dealing with problems well	0.06	−0.03	**0.65**	0.09	0.01	0.10
SWEMWBS 5 I’ve been thinking clearly	0.11	0.00	**0.58**	0.14	−0.05	0.16
SWEMWBS 6 I’ve been feeling close to other people	0.09	−0.06	**0.43**	0.12	0.25	−0.14
SWEMWBS 7 I’ve been able to make up my own mind about things	0.18	−0.05	**0.42**	0.13	0.09	0.08
DASS 1 I found it hard to wind down	0.00	0.06	0.14	0.00	**0.56**	0.10
DASS 2 I was aware of dryness of my mouth	0.06	0.01	−0.02	0.03	−0.01	**0.45**
DASS 3 I couldn’t seem to experience any positive feeling at all	**0.50**	−0.02	0.14	0.12	0.13	0.07
DASS 4 I experienced breathing difficulty	−0.06	0.08	0.09	−0.13	0.02	**0.66**
DASS 5 I found it difficult to work up the initiative to do things	**0.54**	0.11	−0.04	−0.09	0.04	0.10
DASS 6 I tended to over-react to situations	0.25	−0.06	−0.02	0.14	0.23	0.29
DASS 7 I experienced trembling (e.g., in the hands)	0.01	0.04	−0.02	0.03	0.07	**0.58**
DASS 8 I felt that I was using a lot of nervous energy	0.12	−0.02	−0.05	0.01	**0.32**	**0.41**
DASS 9 I was worried about situations in which I might panic and make a fool of myself	**0.34**	0.10	−0.12	0.02	0.18	0.29
DASS 10 I felt that I had nothing to look forward to	**0.72**	−0.02	0.10	0.04	0.03	0.00
DASS 11 I found myself getting agitated	0.28	−0.04	−0.05	0.11	**0.53**	0.14
DASS 12 I found it difficult to relax	0.12	0.13	0.06	−0.08	**0.69**	0.04
DASS 13 I felt downhearted and blue	**0.61**	0.07	0.06	−0.05	0.09	0.09
DASS 14 I was intolerant of anything that kept me from getting on with what I was doing	0.11	−0.08	0.00	0.29	**0.34**	0.15
DASS 15 I felt I was close to panic	0.16	0.09	0.01	0.05	0.06	**0.58**
DASS 16 I was unable to become enthusiastic about anything	**0.68**	0.03	0.05	−0.02	0.14	0.01
DASS 17 I felt I wasn’t worth much as a person	**0.54**	0.16	0.16	−0.06	0.05	0.11
DASS 18 I felt that I was rather touchy	0.19	−0.10	0.02	0.23	0.25	**0.31**
DASS 19 I was aware of the action of my heart in the absence of physical exertion	−0.03	−0.06	0.10	0.04	−0.01	**0.65**
DASS 20 I felt scared without any good reason	0.23	0.12	0.03	−0.05	−0.05	**0.46**
DASS 21 I felt that life was meaningless	**0.74**	0.05	0.09	0.04	−0.04	0.03

*Note*. Loadings ≥ 0.30 are printed in bold. Factor 1 = depression, Factor 2 = voice hearing experience, Factor 3 = well-being, Factor 4 = voice characteristics, Factor 5 = stress, Factor 6 = states of anxiety and stress.

#### Confirmatory factor analysis.

A six-factor model was tested, in which items with cross-loadings were allowed to load onto both factors. In other words, items 1, 3, 4 and 8 of the HPSVQ were allowed to load onto Factors 2 and 4, item 3 of the SWEMWBS was allowed to load onto Factors 3 and 5, and item 8 of the DASS-21 was allowed to load onto Factors 5 and 6. The model had an acceptable fit, χ2/df = 2.04, TLI = 0.914, CFI = 0.922, RMSEA = 0.051 [90% CI 0.047–0.055], SRMR = 0.044.

## Study 2: PSYRATS-AH, HADS and CHOICE-SF

### Methods

#### Design.

Study 2 used baseline data from two trials, the Mindfulness for Voices trial (M4V [31]) and the Guided Self-help Cognitive Behavioural Intervention for Voices trial (GiVE2, [32]). Datasets were merged to identify the underlying structure of the PSYRATS-AH, HADS and CHOICE-SF. Data were accessed between 2019–2023. Data were anonymised and therefore individual participants could not be identified.

#### Participants.

A total of 187 participants experiencing distressing voices were recruited from mental health NHS services. All participants met diagnostic criteria for Schizophrenia-spectrum or Psychotic disorders (Table 3 and S5 Table in S1 File).

**Table 3:** Demographic information of participants in study 2.

	M4V	GiVE2	Merged sample	t-test/z-test
(*N* = 108)	(*N* = 79)	(*N* = 187)
**M age (*SD*)**	40.84 (11.0)	40.11 (13.0)	40.53 (11.9)	*t*(151) = 0.40, *p* = .688
**M age voice onset (*SD*)**	23.90 (10.9)	22.02 (12.0)	23.08 (11.4)	*t*(159.21) = 1.08, *p* = .281
**M voice duration (*SD*)**	16.55 (10.8)	18.09 (13.6)	17.22 (12.08)	*t*(145.63) = −0.83, *p* = .409
**Gender (%)**				
* Male*	53 (49.1)	45 (57.0)	98 (52.4)	*z* = −1.07, *p* = .285
* *Female	54 (50.0)	34 (43.0)	88 (47.1)	*z* = 0.94, *p* = .347
**Ethnicity (%)**				
* White*	102 (94.4)	66 (83.5)	168 (89.8)	*z* = 2.44, *p* = 0.015
* Asian*	3 (2.8)	5 (6.3)	8 (4.3)	*z* = −1.18, *p* = .234
* Black*	2 (1.9)	1 (1.3)	3 (1.6)	*z* = 0.31, *p* = .749
* Mixed*	–	4 (5.1)	4 (2.1)	–
* Other*	1 (0.9)	3 (3.8)	4 (2.1)	*z* = −1.34, *p* = .180
**Employment (%)**				
* Employed*	5 (4.5)	5 (6.3)	10 (5.3)	*z* = −0.51, *p* = .610
* Unemployed*	82 (75.9)	51 (64.5)	133 (71.1)	*z* = 1.69, *p* = .091
* Student*	2 (1.9)	7 (8.9)	9 (4.8)	*z* = −2.21, *p* = .027
* Retired*	4 (3.7)	2 (2.5)	6 (3.2)	*z* = 0.45, *p* = .653
* Homemaker*	3 (2.8)	3 (3.8)	6 (3.2)	*z* = −0.39, *p* = .596
* Other*	12 (11.1)	10 (12.7)	22 (11.8)	*z* = −0.32, *p* = .749
**Education (%)**				
* Left school <16*	30 (27.8)	16 (20.3)	46 (24.6)	*z* = 1.18, *p* = .238
* Left school at 16*	30 (27.8)	28 (35.4)	58 (31.0)	*z* = −1.11, *p* = .263
* Left school at 17/18*	19 (17.6)	6 (7.6)	25 (13.4)	*z* = 1.20, *p* = .048
* College*	17 (15.7)	11 (13.9)	28 (15.0)	*z* = 0.34, *p* = .728
* University*	11 (10.2)	18 (22.8)	29 (15.5)	*z* = −2.35, *p* = .018

*Note*. Merged sample missing data for age voice onset (*n* = 6), voice duration (*n* = 6), gender (*n* = 1), education (*n* = 1) and employment (*n* = 1); M4V sample missing data for age voice onset (*n* = 6), gender (*n* = 1) education (*n* = 1); GiVE2 missing data for employment (*n* = 1).

#### Materials.

**Psychotic Symptom Rating Scales – Auditory Hallucinations (PSYRATS-AH [7]).** The PSYRATS-AH is a structured interview assessing the severity of a variety of dimensions of the voice hearing experience. It consists of 11 items and each one is rated on a scale of 0 (*absent*) to 4 (*severe*). The PSYRATS-AH has been found to have good to excellent inter-rater reliability [7,33] and good internal consistency [33] in schizophrenia and first-episode psychosis populations.

**Hospital Anxiety Depression Scale (HADS [5]).** The HADS is a 14-item self-report questionnaire used to assess anxiety (7 items) and depression (7 items) symptoms. Each item is rated on a 4-point rating scale, with higher scores reflecting more severe symptoms. The HADS has shown good internal consistency in a schizophrenia population [34].

**CHoice *of* Outcome *in* Cbt *for* psychoses – Short Form (CHOICE [14,15]).** The CHOICE-SF is a self-report 11-item questionnaire measuring service-user defined recovery in relation to Cognitive Behavioural Therapy for psychosis (CBTp). The CHOICE has good test-retest reliability and internal consistency [14]. The short form has been validated in a transdiagnostic sample of voice-hearers [15]. All items have been reverse-coded, thereby higher scores on the scale indicate lower recovery.

### Procedure

M4V participants were recruited from mental health services in Sussex and Hampshire, and GiVE2 participants were recruited from sites within Sussex Partnership NHS Foundation Trust and Pennine Care NHS Foundation Trust. In both studies, eligible service users were referred by clinicians. Following written informed consent, participants were asked to complete a list of measures. M4V and GiVE2 were trials of Group Person-Based Cognitive Therapy (PBCT) and Guided self-help cognitive Behavioural intervention for VoicEs (GiVE), respectively. The M4V study (recruitment start date: 03/10/2011, recruitment end date: 02/10/2013) received ethical approval by the Brighton and Sussex REC (11/L0/1330). The GiVE2 study (recruitment start date: 02/01/2019, recruitment end date: 31/08/2020) received ethical approval by the London – Surrey REC (8/LO/ 2091).

### Statistical analysis

Missing values (0.23%) were handled using FIML. Parallel analysis (Horn, 1965) and a scree plot were used to determine the number of factors to retain. Exploratory factor analysis (EFA) with oblique rotation was performed to investigate the underlying factor structure of PSYRATS-AH, HADS and CHOICE-SF. Model fit was determined in the same way as for study 1.

### Results

A Kaiser-Meyer-Olkin statistic indicated adequate numbers of variables to determine a factor structure, KMO = 0.86. Barlett’s test confirmed that correlations were significantly different to zero χ^2^(630) = 2564.94, *p* < .001. Items did not strongly correlate with each other (S6 Tables in S1 File). Parallel analysis suggested five factors to be retained, whilst the scree plot indicated points of inflexion at 2, 3, 4 and 5 factors. The five-factor model was the best fit of the data (TLI = 0.915, RMSEA = 0.037, RMSR = 0.04, CFI = 0.939) (S7 Table in S1 File).

The model accounted for 39.99% of the total variance (Table 4). Factor 1 (psychological recovery) accounted for 15.52% of the variance and consisted of seven items of the HADS relating to depression (anhedonia – items 3, 6, 10 and 14], slowed down – item 2, laugh – item 7, cheerful – item 11) and two relating to anxiety (tension – item 1, feeling relaxed – item 13), and seven items of the CHOICE-SF (ability to approach problems – item 1, self-confidence – item 2, positive social relating – item 3, ability to question way of thinking – item 4, dealing with stresses – item 5, peace of mind – item 8, positive ways of thinking – item 11). Factor 2 (voice impact and phenomenology) consisted of six items of the PSYRATS-AH (duration – item 2, loudness – item 4, amount of negative content – item 6, degree of negative content – item 7, amount of distress – item 8, intensity of distress – item 9) and accounted for 7.47% of the variance. Factor 3 (anxiety) consisted of six items of the HADS (feelings of tension – item 1, fright – items 4 and 5, restlessness – item 8, worry – item 9, panic – item 12) and accounted for 6.53% of variance. Factor 4 (cognitive processing) consisted of three items of the CHOICE (facing upsetting thoughts – item 7, understanding self – item 9, understanding experiences – item 10) and accounted for 6.70% of the variance. Factor 5 (voice frequency) accounted for 3.78% of the variance and consisted of four items (frequency – item 1, duration – item 2, disruption – item 10, controllability – item 11). Items 3 (location) and 5 (beliefs re:origin) of the PSYRATS-AH did not significantly load onto any factor, although there was some indication that item 3 loaded onto Factor 5 and item 5 loaded onto Factor 3. The inter-factor correlations demonstrated small-to-medium correlations between voice-specific (2, 5) and broad factors (1, 3, 4) (S8 Table in S1 File).

**Table 4:** Exploratory factor analysis (PSYRATS-AH, HADS and CHOICE-SF).

Items	F1	F2	F3	F4	F5
PSYRATS 1: Frequency	0.12	0.29	−0.10	−0.09	**0.51**
PSYRATS 2: Duration	0.08	**0.46**	−0.02	−0.06	**0.34**
PSYRATS 3: Location	−0.17	0.16	0.07	−0.18	0.28
PSYRATS 4: Loudness	−0.02	**0.37**	0.05	0.10	0.03
PSYRATS 5: Beliefs re:origin	0.08	0.05	−0.26	0.06	0.19
PSYRATS 6: Amount of negative content	0.03	**0.68**	0.13	−0.07	0.06
PSYRATS 7: Degree of negative content	−0.04	**0.82**	−0.06	−0.02	−0.13
PSYRATS 8: Amount of distress	0.01	**0.58**	0.08	0.15	0.15
PSYRATS 9: Intensity of distress	0.13	**0.51**	0.08	0.09	0.29
PSYRATS 10: Disruption to life	0.15	0.19	−0.08	0.14	**0.31**
PSYRATS 11: Controllability of voices	0.16	−0.05	0.13	0.27	**0.35**
HADS 1: I feel tense or wound up	**0.46**	−0.13	**0.42**	−0.06	0.09
HADS 2: I feel as if I am slowed down	**0.42**	−0.13	0.24	0.05	0.04
HADS 3: I still enjoy the things I used to enjoy	**0.54**	0.10	−0.10	0.14	−0.07
HADS 4: I get a sort of frightened feeling like ‘butterflies’ in my stomach	0.05	0.03	**0.48**	0.05	−0.07
HADS 5: I get a sort of frightened feeling as if something awful is about to happen	0.07	−0.01	**0.53**	0.17	0.06
HADS 6: I have lost interest in my appearance	**0.52**	−0.10	0.02	0.03	0.01
HADS 7: I can laugh and see the funny side of things	**0.50**	0.01	−0.04	0.12	0.06
HADS 8: I feel restless as I have to be on the move	−0.28	0.00	**0.35**	0.22	0.23
HADS 9: Worrying thoughts go through my mind	0.08	0.20	**0.53**	−0.09	−0.23
HADS 10: I look forward with enjoyment to things	**0.67**	−0.03	−0.12	0.11	0.05
HADS 11: I feel cheerful	**0.74**	0.07	−0.14	−0.05	0.09
HADS 12: I get sudden feelings of panic	−0.02	0.08	**0.66**	0.03	0.03
HADS 13: I can sit at ease and feel relaxed	**0.53**	0.09	0.13	0.01	0.11
HADS 14: I can enjoy a good book or radio or TV programme	**0.38**	0.04	−0.04	−0.08	−0.09
CHOICE 1: The ability to approach problems in a variety of ways	**0.35**	0.24	−0.02	0.28	−0.26
CHOICE 2: Self-confidence	**0.60**	0.12	0.14	−0.01	−0.10
CHOICE 3: Positive ways of relating to people	**0.64**	−0.03	0.09	−0.04	0.03
CHOICE 4: The ability to question the way I look at things	**0.41**	0.16	−0.08	0.22	−0.24
CHOICE 5: Ways of dealing with everyday life stresses	**0.49**	0.06	0.23	0.09	−0.16
CHOICE 6: Ways of dealing with a crisis	0.27	−0.01	0.25	0.21	−0.23
CHOICE 7: Facing my own upsetting thoughts and feelings	0.26	0.01	0.07	**0.51**	−0.14
CHOICE 8: Peace of Mind	**0.61**	−0.01	0.21	0.06	0.12
CHOICE 9: Understanding myself and my past	−0.02	−0.01	−0.03	**0.81**	0.08
CHOICE 10: Understanding my experiences	0.00	−0.01	0.04	**0.69**	−0.05
CHOICE 11: Positive ways of thinking	**0.52**	0.06	0.16	0.24	−0.22

*Note*. Loadings ≥ 0.30 are printed in bold. Factor 1 = psychological recovery, Factor 2 = impact and phenomenology of voices, Factor 3 = Worry, Factor 4 = cognitive processing, Factor 5 = Voice frequency.

## Discussion

### Main findings

The current studies aimed to investigate whether voice hearing measures are distinct from broader outcome measures. In Study 1, the factors generated did not contain items from both the HPSVQ and the DASS-21 or from both the HPSVQ and the SWEMWBS. Similarly, in Study 2, the factors generated did not contain items from both the PSYRATS-AH and the HADS or from both the PSYRATS-AH and CHOICE-SF. The HPSVQ and the PSYRATS-AH each showed a two-factor solution. These findings highlight the distinctiveness of voice hearing measures from measures of emotional states, well-being and recovery. While previous research was suggestive of links between voice hearing and broader measures of outcome [2,11–14,17], the current findings suggest these measures are empirically distinct and do not reflect substantial measurement overlap. However, it is worth noting that across both samples, self-rated measures tended to show stronger intercorrelations with each other in comparison to the clinician-rated PSYRATS-AH. This suggests that the rating method may have influenced the observed relationships between measures.

Within Study 1, most of the items of the HPSVQ loaded onto Factor 2. However, the items measuring frequency, loudness, duration, clarity and obey commands also loaded onto Factor 4, forming a factor that resembles the ‘physical’ characteristics’ sub-scale from previous psychometric evaluations., These findings suggest that, although the ‘negative impact’ and the ‘physical characteristics’ sub-scales can overlap, they are, to some extent separate and distinct features of the voice hearing experience. This is in line with previous research, showing that the HPSVQ provides a two-factor solution: emotional and physical characteristics [35,36]. Contrary to previous research demonstrating a three- or four-factor structure for the PSYRATS-AH [7,37–39], our study identified a two-factor solution; Factor 2 (voice impact and phenomenology) consisted of items relating to voice-related distress and the ‘physical’ characteristics of the voices and Factor 5 (voice frequency) consisted of items relating to both the ‘cognitive’ and ‘physical’ characteristics of the voices. The amount and degree of negative content and the amount and intensity of distress loaded onto Factor 2 and the frequency and duration of voices loaded onto Factor 5, consistent with previous studies demonstrating that these items are grouped under the same factors, whilst the remaining items loaded onto various factors across studies [7,37–41].

Differences in findings within the current study could be attributed to the small sample size in study 2; therefore, results should be interpreted with caution. Several items such as items 3 (location) and 5 (beliefs re:origin) of the PSYRATS-AH did not significantly load onto any factor (the general rule of thumb is 0.30 but this can vary depending on sample size [42]. Differences in findings could also be attributed to participant characteristics and a lack of understanding of the dimensions of the voice hearing experience [37,38]. Items from the DASS-21 and the CHOICE-SF appeared to load onto the same factor (Factor 1 psychological recovery), suggesting that subjective recovery as measured by the CHOICE-SF largely captures emotional distress.

The findings provide empirical support for the view that the ‘physical’ characteristics of voices and the emotional responses they can evoke are related but separable aspects of the voice hearing experience, consistent with cognitive models of voice hearing [43,44]. They also support the continued use of measures of voice hearing and of broader measures of emotional states, wellbeing and recovery in both research and routine clinical practice, as they appear to capture unique and supplemental information that can assist with obtaining an extensive and nuanced understanding of people’s experiences. In line with the single-symptom approach, outcomes should match the target; our findings suggest that measures of anxiety and depression index general emotional distress and should not be used as proxies for voice-related distress. Instead, voice hearing measures should be used to assess associated distress, with measures of anxiety, depression, recovery and wellbeing used alongside to capture emotional states and broader life outcomes.

Previous research suggests that voices may become less emotionally salient over time [45,46]. In both studies, the mean participant age was 40 years, and the mean voice hearing duration was 17 years. Therefore, it is possible that our findings were influenced by voice hearing duration. For instance, some dimensions of voice hearing may show greater overlap with emotional distress earlier on in the course, with this overlap attenuating at longer durations. Our analyses focused on measurement structure using EFA in cross-sectional data, thus within-person change cannot be inferred. Future research (ideally longitudinal and sufficiently powered) should model duration continuously and evaluate changes in shared variance over time, and/or examine whether the factor structures replicate in samples with shorter voice hearing duration. Such evidence would help identify when, and for whom, streamlining outcome measures is appropriate.

Our results also highlight several other implications. Firstly, it may be useful to clearly specify the focus of questions, particularly for self-reported measures, as items can be interpreted differently. For example, instructions could indicate whether distress ratings should reflect the impact of voices specifically or mood more generally. Secondly, voice-hearing and broad outcome domain scores are best interpreted together rather than in isolation, as they can help to contextualise and make sense of people’s experience, including how these may influence each other. For example, previous research has shown cross-sectional associations between emotional distress, voice-related beliefs, and voice-related distress [47]. While causality cannot be established, it is plausible that mood and anxiety symptoms may maintain and/or exacerbate voice-related beliefs and associated distress. By considering the broader context, this can help clinicians and researchers to better understand people’s experiences and tailor interventions accordingly.

## Strengths and limitations

Analyses considered whether voice hearing measures were distinct from broad outcome measures using different instruments and datasets. In study 1, two large samples were used with relatively low missing data to identify and to confirm the underlying factor structure, which ensured the reduction of the sampling error, leading to more stable factors. However, despite the Kaiser-Meyer-Olkin statistic indicating that the sample in study 2 was suitable for factor analysis, the size of the sample was small and consequently this may have affected the factor structure by increasing the likelihood of false negatives. A large sample size is needed to undertake EFA to ensure that there is adequate statistical power [48–50]. It was also not possible to confirm the factor structure in study 2 as there was insufficient data to undertake CFA. A further limitation refers to demographic differences between the samples (e.g., the Approve sample was transdiagnostic, whereas participants in the VIS trial met criteria for schizophrenia- or psychotic-spectrum disorders).

## Conclusions

This study found that voice hearing measures were distinct from measures of emotional states, well-being and recovery, suggesting that measures of voice hearing and measures of broader outcomes may not be measuring similar concepts. This confirms the psychometric properties of the PSYRATS-AH and HPSVQ by establishing their distinctiveness from broad outcome measures and encourages their future use as measures of voice hearing experiences.

## Supporting information

S1 File S1 Appendix (DOCX)
