# Supplementary material for: Are measures of voice hearing distinct from measures of emotional states, recovery and well-being? A factor analysis study
Source: PLoS One. 2025 Oct 7;20(10):e0333069. doi: 10.1371/journal.pone.0333069 (PMC12503322; doi:10.1371/journal.pone.0333069)
Supplement: S1 File — (DOCX) [file pone.0333069.s001.docx]

**Table S1.** Descriptive statistics for all HPSVQ, DASS-21 and SWEMWBS items

|  | **[anonymised study 1[ sample** | | | **[anonymised study 2[ sample** | | |
| --- | --- | --- | --- | --- | --- | --- |
| **Items** | ***M*** | ***SD*** | ***N*** | ***M*** | ***SD*** | ***N*** |
| HPSVQ 1 How frequently did you hear a voice or voices? | 2.5 | 1.2 | 401 | 2.7 | 1.2 | 390 |
| HPSVQ 2 How bad are the things the voices say to you? | 2.4 | 1.4 | 399 | 2.4 | 1.4 | 392 |
| HPSVQ 3 How loud are the voices? | 2.2 | 1.1 | 398 | 2.2 | 1.1 | 388 |
| HPSVQ 4 How long do the voices usually last? | 2.7 | 1.3 | 398 | 2.7 | 1.3 | 391 |
| HPSVQ 5 How much do the voices interfere with your daily activities? | 2.1 | 1.3 | 400 | 2.3 | 1.3 | 393 |
| HPSVQ 6 How distressing are the voices that you hear? | 2.5 | 1.4 | 400 | 2.5 | 1.3 | 388 |
| HPSVQ 7 How bad do the voices make you feel about yourself? | 2.2 | 1.4 | 400 | 2.2 | 1.3 | 393 |
| HPSVQ 8 How clearly do you hear the voices? | 3.0 | 1.2 | 400 | 3.0 | 1.1 | 388 |
| HPSVQ 9 How often do you do what the voices say? | 1.3 | 1.2 | 400 | 1.3 | 1.1 | 388 |
| SWEMWBS 1 I’ve been feeling optimistic about the future | 3.2 | 1.1 | 400 | 3.2 | 1.1 | 390 |
| SWEMWBS 2 I’ve been feeling useful | 3.4 | 1.0 | 400 | 3.3 | 1.1 | 389 |
| SWEMWBS 3 I’ve been feeling relaxed | 3.4 | 1.0 | 398 | 3.3 | 1.0 | 387 |
| SWEMWBS 4 I’ve been dealing with problems well | 3.1 | 1.1 | 398 | 3.1 | 1.1 | 390 |
| SWEMWBS 5 I’ve been thinking clearly | 3.0 | 1.1 | 399 | 3.1 | 1.0 | 387 |
| SWEMWBS 6 I’ve been feeling close to other people | 3.0 | 1.2 | 400 | 3.2 | 1.1 | 389 |
| SWEMWBS 7 I’ve been able to make up my own mind about things | 2.6 | 1.1 | 400 | 2.7 | 1.1 | 387 |
| DASS 1 I found it hard to wind down | 1.7 | 1.0 | 399 | 1.6 | 1.0 | 394 |
| DASS 2 I was aware of dryness of my mouth | 1.5 | 1.1 | 399 | 1.4 | 1.1 | 395 |
| DASS 3 I couldn’t seem to experience any positive feeling at all | 1.4 | 1.0 | 397 | 1.4 | 1.0 | 388 |
| DASS 4 I experienced breathing difficulty | 1.1 | 1.1 | 399 | 1.1 | 1.0 | 393 |
| DASS 5 I found it difficult to work up the initiative to do things | 1.7 | 1.0 | 398 | 1.7 | 1.0 | 393 |
| DASS 6 I tended to over-react to situations | 1.3 | 1.1 | 398 | 1.4 | 1.1 | 392 |
| DASS 7 I experienced trembling (e.g., in the hands) | 1.2 | 1.1 | 397 | 1.1 | 1.1 | 395 |
| DASS 8 I felt that I was using a lot of nervous energy | 1.5 | 1.1 | 398 | 1.4 | 1.0 | 393 |
| DASS 9 I was worried about situations in which I might panic and make a fool of myself | 1.6 | 1.1 | 398 | 1.5 | 1.1 | 393 |
| DASS 10 I felt that I had nothing to look forward to | 1.4 | 1.1 | 399 | 1.5 | 1.1 | 394 |
| DASS 11 I found myself getting agitated | 1.7 | 1.0 | 398 | 1.7 | 1.0 | 390 |
| DASS 12 I found it difficult to relax | 1.8 | 1.0 | 399 | 1.8 | 1.0 | 393 |
| DASS 13 I felt downhearted and blue | 1.6 | 1.0 | 399 | 1.6 | 1.0 | 391 |
| DASS 14 I was intolerant of anything that kept me from getting on with what I was doing | 1.2 | 1.0 | 395 | 1.2 | 1.0 | 381 |
| DASS 15 I felt I was close to panic | 1.3 | 1.1 | 398 | 1.3 | 1.0 | 385 |
| DASS 16 I was unable to become enthusiastic about anything | 1.4 | 1.1 | 398 | 1.4 | 1.0 | 384 |
| DASS 17 I felt I wasn’t worth much as a person | 1.6 | 1.1 | 396 | 1.5 | 1.1 | 385 |
| DASS 18 I felt that I was rather touchy | 1.3 | 1.0 | 397 | 1.3 | 1.0 | 386 |
| DASS 19 I was aware of the action of my heart in the absence of physical exertion | 1.2 | 1.1 | 397 | 1.1 | 1.0 | 386 |
| DASS 20 I felt scared without any good reason | 1.4 | 1.1 | 399 | 1.4 | 1.1 | 383 |
| DASS 21 I felt that life was meaningless | 1.3 | 1.1 | 397 | 1.3 | 1.1 | 386 |

**Table S2.** Correlation matrix of all HPSVQ, DASS-21 and SWEMWBS items

| **Items** | **1** | **2** | **3** | **4** | **5** | **6** | **7** | **8** | **9** | **10** | **11** | **12** | **13** | **14** | **15** | **16** | **17** | **18** | **19** | **20** | **21** | **22** | **23** | **24** | **25** | **26** | **27** | **28** | **29** | **30** | **31** | **32** | **33** | **34** | **35** | **36** | **37** |
| --- | --- | --- | --- | --- | --- | --- | --- | --- | --- | --- | --- | --- | --- | --- | --- | --- | --- | --- | --- | --- | --- | --- | --- | --- | --- | --- | --- | --- | --- | --- | --- | --- | --- | --- | --- | --- | --- |
| **1. HPSVQ 1** | 1 |  |  |  |  |  |  |  |  |  |  |  |  |  |  |  |  |  |  |  |  |  |  |  |  |  |  |  |  |  |  |  |  |  |  |  |  |
| **2. HPSVQ 2** | .48 | 1 |  |  |  |  |  |  |  |  |  |  |  |  |  |  |  |  |  |  |  |  |  |  |  |  |  |  |  |  |  |  |  |  |  |  |  |
| **3. HPSVQ 3** | .53 | .50 | 1 |  |  |  |  |  |  |  |  |  |  |  |  |  |  |  |  |  |  |  |  |  |  |  |  |  |  |  |  |  |  |  |  |  |  |
| **4. HPSVQ 4** | .62 | .49 | .53 | 1 |  |  |  |  |  |  |  |  |  |  |  |  |  |  |  |  |  |  |  |  |  |  |  |  |  |  |  |  |  |  |  |  |  |
| **5. HPSVQ 5** | .52 | .61 | .55 | .59 | 1 |  |  |  |  |  |  |  |  |  |  |  |  |  |  |  |  |  |  |  |  |  |  |  |  |  |  |  |  |  |  |  |  |
| **6. HPSVQ 6** | .47 | .75 | .61 | .57 | .69 | 1 |  |  |  |  |  |  |  |  |  |  |  |  |  |  |  |  |  |  |  |  |  |  |  |  |  |  |  |  |  |  |  |
| **7. HPSVQ 7** | .40 | .68 | .56 | .51 | .66 | .72 | 1 |  |  |  |  |  |  |  |  |  |  |  |  |  |  |  |  |  |  |  |  |  |  |  |  |  |  |  |  |  |  |
| **8. HPSVQ 8** | .54 | .48 | .58 | .59 | .47 | .54 | .47 | 1 |  |  |  |  |  |  |  |  |  |  |  |  |  |  |  |  |  |  |  |  |  |  |  |  |  |  |  |  |  |
| **9. HPSVQ 9** | .42 | .31 | .42 | .42 | .41 | .36 | .38 | .37 | 1 |  |  |  |  |  |  |  |  |  |  |  |  |  |  |  |  |  |  |  |  |  |  |  |  |  |  |  |  |
| **11. SWEMWBS 1** | .13 | .24 | .24 | .18 | .30 | .29 | .34 | .12 | .28 | 1 |  |  |  |  |  |  |  |  |  |  |  |  |  |  |  |  |  |  |  |  |  |  |  |  |  |  |  |
| **11. SWEMWBS 2** | .10 | .21 | .18 | .19 | .29 | .26 | .31 | .09 | .18 | .55 | 1 |  |  |  |  |  |  |  |  |  |  |  |  |  |  |  |  |  |  |  |  |  |  |  |  |  |  |
| **12. SWEMWBS 3** | .24 | .32 | .30 | .27 | .40 | .38 | .43 | .22 | .24 | .49 | .49 | 1 |  |  |  |  |  |  |  |  |  |  |  |  |  |  |  |  |  |  |  |  |  |  |  |  |  |
| **13. SWEMWBS 4** | .18 | .26 | .29 | .22 | .32 | .31 | .35 | .15 | .33 | .56 | .49 | .47 | 1 |  |  |  |  |  |  |  |  |  |  |  |  |  |  |  |  |  |  |  |  |  |  |  |  |
| **14. SWEMWBS 5** | .23 | .25 | .29 | .29 | .36 | .33 | .41 | .21 | .39 | .50 | .42 | .50 | .62 | 1 |  |  |  |  |  |  |  |  |  |  |  |  |  |  |  |  |  |  |  |  |  |  |  |
| **15. SWEMWBS 6** | .17 | .16 | .25 | .17 | .31 | .18 | .25 | .13 | .18 | .35 | .37 | .35 | .36 | .39 | 1 |  |  |  |  |  |  |  |  |  |  |  |  |  |  |  |  |  |  |  |  |  |  |
| **16. SWEMWBS 7** | .21 | .23 | .26 | .19 | .29 | .30 | .33 | .14 | .32 | .44 | .38 | .42 | .47 | .52 | .41 | 1 |  |  |  |  |  |  |  |  |  |  |  |  |  |  |  |  |  |  |  |  |  |
| **17. DASS 1** | .21 | .27 | .31 | .24 | .35 | .31 | .35 | .17 | .21 | .32 | .31 | .50 | .34 | .34 | .31 | .36 | 1 |  |  |  |  |  |  |  |  |  |  |  |  |  |  |  |  |  |  |  |  |
| **18. DASS 2** | .10 | .16 | .18 | .13 | .17 | .22 | .22 | .14 | .19 | .18 | .12 | .27 | .20 | .20 | .13 | .22 | .23 | 1 |  |  |  |  |  |  |  |  |  |  |  |  |  |  |  |  |  |  |  |
| **19. DASS 3** | .25 | .23 | .32 | .30 | .41 | .35 | .41 | .15 | .35 | .45 | .42 | .43 | .46 | .47 | .35 | .41 | .45 | .28 | 1 |  |  |  |  |  |  |  |  |  |  |  |  |  |  |  |  |  |  |
| **20. DASS 4** | .10 | .17 | .30 | .14 | .29 | .32 | .32 | .09 | .22 | .31 | .27 | .37 | .31 | .34 | .18 | .26 | .38 | .31 | .36 | 1 |  |  |  |  |  |  |  |  |  |  |  |  |  |  |  |  |  |
| **21. DASS 5** | .18 | .27 | .24 | .17 | .36 | .34 | .43 | .14 | .27 | .40 | .38 | .35 | .38 | .43 | .29 | .35 | .35 | .22 | .49 | .44 | 1 |  |  |  |  |  |  |  |  |  |  |  |  |  |  |  |  |
| **22. DASS 6** | .14 | .25 | .26 | .21 | .31 | .28 | .32 | .12 | .32 | .27 | .24 | .32 | .38 | .36 | .23 | .33 | .43 | .33 | .44 | .36 | .38 | 1 |  |  |  |  |  |  |  |  |  |  |  |  |  |  |  |
| **23. DASS 7** | .19 | .21 | .22 | .25 | .30 | .28 | .28 | .17 | .18 | .25 | .22 | .33 | .27 | .29 | .19 | .25 | .33 | .38 | .39 | .41 | .28 | .37 | 1 |  |  |  |  |  |  |  |  |  |  |  |  |  |  |
| **24. DASS 8** | .17 | .24 | .20 | .20 | .29 | .28 | .30 | .12 | .20 | .26 | .24 | .39 | .32 | .34 | .25 | .33 | .45 | .30 | .47 | .42 | .34 | .44 | .46 | 1 |  |  |  |  |  |  |  |  |  |  |  |  |  |
| **25. DASS 9** | .22 | .31 | .25 | .26 | .39 | .34 | .39 | .18 | .28 | .27 | .25 | .40 | .34 | .34 | .20 | .37 | .39 | .25 | .42 | .36 | .46 | .50 | .41 | .45 | 1 |  |  |  |  |  |  |  |  |  |  |  |  |
| **26. DASS 10** | .18 | .22 | .26 | .20 | .36 | .33 | .41 | .17 | .31 | .49 | .42 | .41 | .45 | .47 | .37 | .45 | .37 | .33 | .58 | .37 | .52 | .43 | .31 | .43 | .46 | 1 |  |  |  |  |  |  |  |  |  |  |  |
| **27. DASS 11** | .26 | .25 | .30 | .27 | .34 | .34 | .37 | .18 | .22 | .30 | .34 | .45 | .38 | .38 | .32 | .39 | .50 | .25 | .52 | .38 | .44 | .52 | .40 | .54 | .49 | .53 | 1 |  |  |  |  |  |  |  |  |  |  |
| **28. DASS 12** | .22 | .32 | .33 | .27 | .42 | .39 | .44 | .22 | .23 | .35 | .35 | .60 | .37 | .38 | .36 | .40 | .64 | .28 | .49 | .40 | .47 | .43 | .34 | .53 | .49 | .47 | .67 | 1 |  |  |  |  |  |  |  |  |  |
| **29. DASS 13** | .19 | .30 | .25 | .25 | .38 | .37 | .45 | .19 | .27 | .49 | .40 | .46 | .47 | .45 | .31 | .42 | .45 | .26 | .62 | .37 | .53 | .43 | .41 | .46 | .47 | .62 | .52 | .53 | 1 |  |  |  |  |  |  |  |  |
| **30. DASS 14** | .25 | .19 | .25 | .28 | .32 | .25 | .22 | .18 | .27 | .19 | .14 | .31 | .27 | .27 | .29 | .33 | .36 | .19 | .39 | .26 | .30 | .35 | .30 | .36 | .35 | .33 | .44 | .42 | .34 | 1 |  |  |  |  |  |  |  |
| **31. DASS 15** | .28 | .32 | .33 | .30 | .39 | .40 | .45 | .23 | .34 | .33 | .30 | .43 | .41 | .47 | .24 | .43 | .42 | .31 | .48 | .54 | .47 | .45 | .49 | .52 | .55 | .48 | .54 | .53 | .54 | .40 | 1 |  |  |  |  |  |  |
| **32. DASS 16** | .22 | .23 | .28 | .20 | .39 | .35 | .42 | .16 | .29 | .47 | .44 | .47 | .44 | .46 | .33 | .43 | .41 | .28 | .59 | .39 | .64 | .43 | .36 | .44 | .50 | .63 | .56 | .55 | .64 | .35 | .51 | 1 |  |  |  |  |  |
| **33. DASS 17** | .25 | .33 | .34 | .30 | .42 | .42 | .58 | .22 | .35 | .49 | .51 | .51 | .46 | .51 | .35 | .47 | .48 | .32 | .59 | .42 | .54 | .43 | .39 | .45 | .49 | .63 | .48 | .57 | .62 | .31 | .56 | .64 | 1 |  |  |  |  |
| **34. DASS 18** | .24 | .21 | .32 | .22 | .28 | .28 | .31 | .21 | .31 | .25 | .32 | .34 | .33 | .37 | .26 | .36 | .44 | .29 | .44 | .35 | .36 | .52 | .35 | .41 | .40 | .42 | .54 | .46 | .41 | .39 | .47 | .44 | .47 | 1 |  |  |  |
| **35. DASS 19** | .14 | .09 | .20 | .18 | .23 | .23 | .24 | .14 | .25 | .30 | .27 | .35 | .28 | .29 | .20 | .29 | .30 | .35 | .37 | .48 | .34 | .31 | .40 | .39 | .33 | .32 | .38 | .38 | .40 | .29 | .48 | .37 | .41 | .42 | 1 |  |  |
| **36. DASS 20** | .15 | .27 | .23 | .23 | .36 | .36 | .40 | .16 | .27 | .32 | .28 | .36 | .32 | .42 | .20 | .39 | .32 | .26 | .42 | .40 | .39 | .30 | .37 | .43 | .46 | .42 | .42 | .42 | .47 | .26 | .55 | .43 | .49 | .43 | .43 | 1 |  |
| **37. DASS 21** | .23 | .31 | .29 | .26 | .38 | .38 | .46 | .20 | .37 | .52 | .43 | .42 | .44 | .49 | .36 | .50 | .40 | .32 | .59 | .35 | .51 | .44 | .36 | .42 | .48 | .67 | .48 | .51 | .68 | .33 | .50 | .62 | .72 | .44 | .37 | .49 | 1 |

**Table S3.** Fit indices for each model (HPSVQ, DASS-21 and SWEMWBS)

| **Models** | **RMSEA** | **RMSR** | **TLI** | **CFI** |
| --- | --- | --- | --- | --- |
| 2-factor model | 0.069 [0.065, 0.073] | 0.05 | 0.837 | 0.856 |
| 3-factor model | 0.059 [0.055, 0.063] | 0.04 | 0.882 | 0.902 |
| 4-factor model | 0.052 [0.048, 0.057] | 0.03 | 0.906 | 0.927 |
| 5-factor model | 0.046 [0.041, 0.050] | 0.03 | 0.928 | 0.948 |
| 6-factor model | 0.037 [0.031, 0.042] | 0.02 | 0.953 | 0.968 |

**Table S4.** Factor correlations (HPSVQ, DASS-21 and SWEMWBS)

|  | **Factor 1** | **Factor 2** | **Factor 3** | **Factor 4** | **Factor 5** | **Factor 6** |
| --- | --- | --- | --- | --- | --- | --- |
| **Factor 1** | 1 |  |  |  |  |  |
| **Factor 2** | .36 | 1 |  |  |  |  |
| **Factor 3** | .62 | .35 | 1 |  |  |  |
| **Factor 4** | .12 | .36 | .11 | 1 |  |  |
| **Factor 5** | .52 | .31 | .38 | .13 | 1 |  |
| **Factor 6** | .62 | .34 | .43 | .15 | .54 | 1 |

*Note*. Factor 1 = depression, Factor 2 = voice hearing experience, Factor 3 = well-being, Factor 4 = voice characteristics, Factor 5 = stress, Factor 6 = states of anxiety and stress

**Table S5.** Descriptive statistics of all PSYRATS, HADS and CHOICE-SF items

| **Items** | ***M*** | ***SD*** | ***N*** |
| --- | --- | --- | --- |
| PSYRATS 1 Frequency | 2.7 | 1.2 | 187 |
| PSYRATS 2 Duration | 3.0 | 1.1 | 187 |
| PSYRATS 3 Location | 2.6 | 1.2 | 186 |
| PSYRATS 4 Loudness | 2.4 | 1.1 | 187 |
| PSYRATS 5 Beliefs re:origin | 2.1 | 1.2 | 183 |
| PSYRATS 6 Amount of negative content | 3.1 | 1.0 | 186 |
| PSYRATS 7 Degree of negative content | 3.2 | 1.0 | 185 |
| PSYRATS 8 Amount of distress | 3.1 | 0.9 | 187 |
| PSYRATS 9 Intensity of distress | 2.8 | 1.0 | 186 |
| PSYRATS 10 Disruption to life | 1.7 | 0.7 | 185 |
| PSYRATS 11 Controllability of voices | 3.0 | 1.1 | 185 |
| HADS 1 I feel tense or wound up | 1.9 | 0.9 | 187 |
| HADS 2 I feel as if I am slowed down | 1.8 | 1.0 | 187 |
| HADS 3 I still enjoy the things I used to enjoy | 1.5 | 0.9 | 187 |
| HADS 4 I get a sort of frightened feeling like ‘butterflies’ in my stomach | 1.6 | 1.0 | 187 |
| HADS 5 I get a sort of frightened feeling as if something awful is about to happen | 2.1 | 1.0 | 187 |
| HADS 6 I have lost interest in my appearance | 1.5 | 1.0 | 186 |
| HADS 7 I can laugh and see the funny side of things | 1.2 | 1.0 | 187 |
| HADS 8 I feel restless as I have to be on the move | 1.7 | 1.0 | 187 |
| HADS 9 Worrying thoughts go through my mind | 2.3 | 0.8 | 187 |
| HADS10 I look forward with enjoyment to things | 1.5 | 1.0 | 187 |
| HADS 11 I feel cheerful | 1.4 | 0.8 | 186 |
| HADS 12 I get sudden feelings of panic | 1.9 | 0.9 | 187 |
| HADS 13 I can sit at ease and feel relaxed | 1.8 | 0.8 | 187 |
| HADS 14 I can enjoy a good book or radio or TV programme | 1.3 | 1.0 | 187 |
| CHOICE 1 The ability to approach problems in a variety of ways | 5.5 | 2.3 | 187 |
| CHOICE 2 Self-confidence | 6.5 | 2.6 | 187 |
| CHOICE 3 Positive ways of relating to people | 5.2 | 2.4 | 187 |
| CHOICE 4 The ability to question the way I look at things | 5.1 | 2.4 | 187 |
| CHOICE 5 Ways of dealing with everyday life stresses | 5.8 | 2.4 | 187 |
| CHOICE 6 Ways of dealing with a crisis | 6.5 | 2.6 | 187 |
| CHOICE 7 Facing my own upsetting thoughts and feelings | 6.5 | 2.3 | 187 |
| CHOICE 8 Peace of Mind | 6.7 | 2.4 | 187 |
| CHOICE 9 Understanding myself and my past | 5.2 | 2.9 | 187 |
| CHOICE 10 Understanding my experiences | 5.3 | 2.7 | 187 |
| CHOICE 11 Positive ways of thinking | 5.9 | 2.5 | 187 |

**Table S6.** Correlation matrix of all PSYRATS-AH, HADS and CHOICE items

| **Items** | **1** | **2** | **3** | **4** | **5** | **6** | **7** | **8** | **9** | **10** | **11** | **12** | **13** | **14** | **15** | **16** | **17** | **18** | **19** | **20** | **21** | **22** | **23** | **24** | **25** | **26** | **27** | **28** | **29** | **30** | **31** | **32** | **33** | **34** | **35** | **36** |
| --- | --- | --- | --- | --- | --- | --- | --- | --- | --- | --- | --- | --- | --- | --- | --- | --- | --- | --- | --- | --- | --- | --- | --- | --- | --- | --- | --- | --- | --- | --- | --- | --- | --- | --- | --- | --- |
| **1. PSYRATS 1** | 1 |  |  |  |  |  |  |  |  |  |  |  |  |  |  |  |  |  |  |  |  |  |  |  |  |  |  |  |  |  |  |  |  |  |  |  |
| **2. PSYRATS 2** | .49 | 1 |  |  |  |  |  |  |  |  |  |  |  |  |  |  |  |  |  |  |  |  |  |  |  |  |  |  |  |  |  |  |  |  |  |  |
| **3. PSYRATS 3** | .15 | .14 | 1 |  |  |  |  |  |  |  |  |  |  |  |  |  |  |  |  |  |  |  |  |  |  |  |  |  |  |  |  |  |  |  |  |  |
| **4. PSYRATS 4** | .13 | .23 | .07 | 1 |  |  |  |  |  |  |  |  |  |  |  |  |  |  |  |  |  |  |  |  |  |  |  |  |  |  |  |  |  |  |  |  |
| **5. PSYRATS 5** | .08 | .12 | .13 | .03 | 1 |  |  |  |  |  |  |  |  |  |  |  |  |  |  |  |  |  |  |  |  |  |  |  |  |  |  |  |  |  |  |  |
| **6. PSYRATS 6** | .27 | .32 | .21 | .27 | -.01 | 1 |  |  |  |  |  |  |  |  |  |  |  |  |  |  |  |  |  |  |  |  |  |  |  |  |  |  |  |  |  |  |
| **7. PSYRATS 7** | .27 | .41 | .15 | .28 | -.01 | .61 | 1 |  |  |  |  |  |  |  |  |  |  |  |  |  |  |  |  |  |  |  |  |  |  |  |  |  |  |  |  |  |
| **8. PSYRATS 8** | .21 | .33 | .11 | .26 | .06 | .49 | .43 | 1 |  |  |  |  |  |  |  |  |  |  |  |  |  |  |  |  |  |  |  |  |  |  |  |  |  |  |  |  |
| **9. PSYRATS 9** | .32 | .41 | .14 | .30 | .15 | .42 | .36 | .61 | 1 |  |  |  |  |  |  |  |  |  |  |  |  |  |  |  |  |  |  |  |  |  |  |  |  |  |  |  |
| **10. PSYRATS 10** | .26 | .24 | .28 | .11 | .17 | .20 | .14 | .19 | .26 | 1 |  |  |  |  |  |  |  |  |  |  |  |  |  |  |  |  |  |  |  |  |  |  |  |  |  |  |
| **11. PSYRATS 11** | .14 | .10 | .20 | .11 | .05 | .15 | -.04 | .21 | .19 | .21 | 1 |  |  |  |  |  |  |  |  |  |  |  |  |  |  |  |  |  |  |  |  |  |  |  |  |  |
| **12. HADS 1** | .06 | .08 | .05 | .05 | -.05 | .12 | .00 | .15 | .19 | .05 | .17 | 1 |  |  |  |  |  |  |  |  |  |  |  |  |  |  |  |  |  |  |  |  |  |  |  |  |
| **13.HADS 2** | .05 | .09 | -.09 | .10 | -.14 | .03 | .03 | .11 | .17 | .05 | .15 | .47 | 1 |  |  |  |  |  |  |  |  |  |  |  |  |  |  |  |  |  |  |  |  |  |  |  |
| **14. HADS 3** | .08 | .10 | .00 | .08 | .04 | .15 | .21 | .19 | .24 | .27 | .16 | .21 | .27 | 1 |  |  |  |  |  |  |  |  |  |  |  |  |  |  |  |  |  |  |  |  |  |  |
| **15. HADS 4** | -.07 | .06 | .01 | .14 | -.09 | .09 | .02 | .08 | .17 | .05 | .12 | .32 | .15 | .20 | 1 |  |  |  |  |  |  |  |  |  |  |  |  |  |  |  |  |  |  |  |  |  |
| **16. HADS 5** | .01 | .05 | .03 | .03 | -.09 | .17 | .03 | .16 | .21 | .06 | .17 | .43 | .29 | .22 | .37 | 1 |  |  |  |  |  |  |  |  |  |  |  |  |  |  |  |  |  |  |  |  |
| **17. HADS 6** | -.04 | .06 | .02 | .10 | .02 | .09 | .04 | .12 | .08 | .07 | .18 | .29 | .35 | .32 | .10 | .24 | 1 |  |  |  |  |  |  |  |  |  |  |  |  |  |  |  |  |  |  |  |
| **18. HADS 7** | .06 | .12 | .02 | .16 | .01 | .12 | .14 | .18 | .29 | .03 | .23 | .23 | .23 | .38 | .22 | .26 | .33 | 1 |  |  |  |  |  |  |  |  |  |  |  |  |  |  |  |  |  |  |
| **19. HADS 8** | .10 | .03 | .09 | .05 | -.09 | .04 | -.02 | .12 | .05 | .06 | .24 | .08 | .07 | -.08 | .02 | .17 | -.07 | -.09 | 1 |  |  |  |  |  |  |  |  |  |  |  |  |  |  |  |  |  |
| **20. HADS 9** | -.09 | .01 | -.01 | .09 | -.12 | .21 | .23 | .22 | .17 | -.06 | .15 | .27 | .25 | .18 | .29 | .32 | .15 | .11 | .12 | 1 |  |  |  |  |  |  |  |  |  |  |  |  |  |  |  |  |
| **21. HADS 10** | .04 | .06 | -.03 | .07 | .08 | .15 | .15 | .21 | .32 | .11 | .26 | .35 | .34 | .53 | .14 | .20 | .36 | .50 | -.10 | .14 | 1 |  |  |  |  |  |  |  |  |  |  |  |  |  |  |  |
| **22. HADS 11** | .14 | .15 | .00 | .09 | .06 | .26 | .20 | .24 | .27 | .20 | .27 | .29 | .22 | .39 | .11 | .16 | .31 | .50 | .13 | .16 | .44 | 1 |  |  |  |  |  |  |  |  |  |  |  |  |  |  |
| **23. HADS 12** | -.05 | .06 | .12 | .12 | -.10 | .19 | .05 | .13 | .22 | .16 | .17 | .32 | .24 | .18 | .46 | .47 | .12 | .17 | .21 | .39 | .12 | .15 | 1 |  |  |  |  |  |  |  |  |  |  |  |  |  |
| **24. HADS 13** | .17 | .18 | -.04 | .19 | -.04 | .19 | .20 | .26 | .33 | .13 | .27 | .37 | .30 | .26 | .19 | .25 | .27 | .36 | .12 | .28 | .41 | .47 | .20 | 1 |  |  |  |  |  |  |  |  |  |  |  |  |
| **25. HADS 14** | -.03 | -.04 | .05 | .06 | -.05 | .17 | .12 | .04 | .02 | .19 | .07 | .12 | .18 | .23 | .06 | .01 | .11 | .15 | .00 | .13 | .24 | .26 | .10 | .27 | 1 |  |  |  |  |  |  |  |  |  |  |  |
| **26. CHOICE 1** | .01 | .12 | -.01 | .22 | .02 | .17 | .26 | .23 | .28 | .18 | .10 | .29 | .24 | .46 | .16 | .18 | .25 | .30 | -.02 | .25 | .35 | .30 | .19 | .29 | .22 | 1 |  |  |  |  |  |  |  |  |  |  |
| **27. CHOICE 2** | -.04 | .12 | -.02 | .16 | -.01 | .30 | .16 | .28 | .31 | .20 | .17 | .41 | .36 | .38 | .21 | .29 | .34 | .34 | -.04 | .27 | .37 | .47 | .27 | .46 | .28 | .43 | 1 |  |  |  |  |  |  |  |  |  |
| **28. CHOICE 3** | .10 | .17 | -.05 | .07 | -.03 | .18 | .07 | .18 | .22 | .14 | .17 | .40 | .34 | .32 | .23 | .24 | .35 | .28 | -.02 | .22 | .38 | .46 | .18 | .38 | .24 | .29 | .49 | 1 |  |  |  |  |  |  |  |  |
| **29. CHOICE 4** | -.02 | .06 | -.11 | .14 | .08 | .17 | .19 | .24 | .15 | .14 | .12 | .26 | .19 | .40 | .16 | .26 | .27 | .20 | -.08 | .20 | .28 | .40 | .12 | .36 | .23 | .49 | .43 | .38 | 1 |  |  |  |  |  |  |  |
| **30. CHOICE 5** | -.01 | .07 | .05 | .20 | -.05 | .18 | .13 | .22 | .15 | .08 | .26 | .41 | .29 | .40 | .28 | .25 | .28 | .29 | .07 | .34 | .39 | .39 | .31 | .42 | .16 | .50 | .51 | .44 | .47 | 1 |  |  |  |  |  |  |
| **31. CHOICE 6** | -.10 | .04 | -.02 | .08 | -.15 | .11 | .03 | .06 | .13 | .03 | .14 | .28 | .21 | .28 | .33 | .26 | .22 | .30 | -.01 | .32 | .29 | .21 | .25 | .29 | .09 | .46 | .36 | .38 | .24 | .50 | 1 |  |  |  |  |  |
| **32. CHOICE 7** | -.06 | .04 | .02 | .11 | -.07 | .13 | .12 | .17 | .15 | .17 | .22 | .27 | .37 | .42 | .26 | .27 | .28 | .29 | .12 | .18 | .36 | .26 | .16 | .34 | .13 | .43 | .41 | .38 | .41 | .42 | .49 | 1 |  |  |  |  |
| **33. CHOICE 8** | .17 | .12 | .09 | .10 | -.01 | .26 | .12 | .22 | .27 | .21 | .29 | .51 | .41 | .40 | .19 | .31 | .35 | .32 | .14 | .29 | .43 | .43 | .25 | .48 | .26 | .44 | .52 | .47 | .30 | .56 | .43 | .47 | 1 |  |  |  |
| **34. CHOICE 9** | .06 | .04 | .09 | .13 | .00 | .06 | .06 | .20 | .16 | .16 | .28 | .15 | .22 | .23 | .15 | .30 | .17 | .24 | .16 | .06 | .30 | .21 | .12 | .24 | .05 | .36 | .26 | .28 | .40 | .26 | .31 | .45 | .30 | 1 |  |  |
| **35. CHOICE 10** | -.14 | .03 | .09 | .10 | -.02 | .07 | .08 | .21 | .20 | .04 | .22 | .26 | .29 | .24 | .15 | .27 | .20 | .31 | .07 | .13 | .30 | .25 | .19 | .24 | .12 | .32 | .24 | .18 | .27 | .23 | .28 | .50 | .25 | .59 | 1 |  |
| **36. CHOICE 11** | -.05 | .05 | .02 | .13 | -.07 | .02 | .02 | .23 | .21 | .10 | .23 | .47 | .42 | .46 | .25 | .35 | .37 | .40 | .01 | .35 | .48 | .45 | .29 | .40 | .21 | .57 | .55 | .44 | .49 | .59 | .46 | .59 | .58 | .37 | .40 | 1 |

**Table S7.** Fit indices for each model (PSYRATS-AH, DASS-21 and CHOICE-SF)

| **Model** | **RMSEA** | **RMSR** | **TLI** | **CFI** |
| --- | --- | --- | --- | --- |
| 2-factor model | 0.059 [0.053, 0.066] | 0.07 | 0.782 | 0.808 |
| 3-factor model | 0.052 [0.045, 0.060] | 0.06 | 0.829 | 0.860 |
| 4-factor model | 0.037 [0.038, 0.054] | 0.05 | 0.869 | 0.900 |
| 5-factor model | 0.037 [0.027, 0.046] | 0.04 | 0.915 | 0.939 |

**Table S8.** Factor correlations (PSYRATS-AH, HADS and CHOICE)

|  | **Factor 1** | **Factor 2** | **Factor 3** | **Factor 4** | **Factor 5** |
| --- | --- | --- | --- | --- | --- |
| **Factor 1** | 1 |  |  |  |  |
| **Factor 2** | .31 | 1 |  |  |  |
| **Factor 3** | .37 | .12 | 1 |  |  |
| **Factor 4** | .48 | .16 | .28 | 1 |  |
| **Factor 5** | -.06 | .13 | -.03 | -.06 | 1 |

*Note*. Factor 1 = psychological recovery, Factor 2 = impact and phenomenology of voices, Factor 3 = Anxiety, Factor 4 = cognitive processing, Factor 5 = voice frequency.
